# The Effects of Acute Sleep Deprivation on Cognitive Control Mechanisms Associated With Hallucinatory Experiences

**DOI:** 10.1111/jsr.70259

**Published:** 2025-12-09

**Authors:** Georgia Punton, Jason G. Ellis, Emily Jensen, Connor Malby, Fatima Sharif, David Smailes, Mark Turnbull, Peter Moseley

**Affiliations:** ^1^ Department of Psychology Northumbria University Newcastle‐upon‐Tyne UK; ^2^ Northumbria Centre for Sleep Research Northumbria University Newcastle‐upon‐Tyne UK; ^3^ Department of Psychology Durham University Durham UK

**Keywords:** inhibition, psychosis, signal detection, sleep health

## Abstract

Sleep dysfunction can impair cognition and may play a causal role in the development of hallucinations. Deficits in cognitive control have been implicated in cognitive models of hallucinations. To better understand the underpinning role of cognition in the relationship between sleep and hallucinations, the current study aimed to investigate the impact of sleep deprivation on cognitive control mechanisms such as intentional inhibition and working memory, as well as auditory signal detection. Forty‐five participants were allocated to either a sleep‐deprivation condition (*N* = 15) or a rested control group condition (*N* = 30). Cognitive control assessments were applied at three timepoints for each condition: baseline (T1), post‐sleep deprivation/post‐habitual sleep (T2), and post‐recovery sleep/post‐habitual daily activity (T3). Results showed significant effects of sleep deprivation on intentional inhibition and working memory, but not auditory signal detection. Findings support current neurocognitive theories and suggest that sleep deprivation may lead to hallucinations through effects on intrusive thoughts and memories. Future research should continue to explore the potential mechanistic pathway between cognitive control and sleep, to inform future intervention work. The study pre‐registration, open materials, data, and code are available on the Open Science Framework (doi.org/10.17605/OSF.IO/28SRW).

## 1 Introduction

Sleep is important for the maintenance of human functioning (Grandner 2017; Weinberg et al. 2016) and cognitive processing (Deak and Stickgold 2010). Poor sleep health (including factors such as sleep loss, sleep disorders, and poor sleep quality) is associated with cognitive impairments in memory, attention, perception, and cognitive control (Deak and Stickgold 2010; García et al. 2021; Kim et al. 2022).

Cognitive control encompasses mechanisms involved in intentional top‐down influence over lower‐level task‐relevant processes, and regulates thoughts, emotions, and behaviours (Miller and Cohen 2001). Selective attention and conscious cognitive inhibition within working memory are key for the effortful selection of appropriate goal‐directed responses (Aron 2007), and higher‐level regulatory processes that enact top‐down control over intrusive thoughts (Pacheco et al. 2020). Sleep deprivation preferentially affects prefrontal areas (Verweij et al. 2014) and differentially affects subcomponents of cognitive control (Kusztor et al. 2019).

Sleep deprivation may also affect perceptual processing (Balter et al. 2022). Perceptual errors are more likely to occur should an individual attend to or fail to inhibit irrelevant or intrusive information in working memory (Allen et al. 2011). Hallucinations amongst both clinical and non‐clinical groups have long been associated with indices of poor sleep (Brederoo et al. 2021; Reeve et al. 2018). A recent review has highlighted potential mechanistic pathways from the well‐established link between dysfunctional sleep and hallucinations (Sheaves et al. 2025), one of which may implicate thought control (Punton et al. 2024). Despite this, there is still little known about the mechanisms underpinning this relationship. Cognitive models of hallucination point to some of the same prefrontal cognitive control mechanisms impaired by sleep deprivation (e.g., Badcock et al. 2007). It therefore seems possible that cognitive control plays a role in the relationship between sleep and hallucinations.

However, when it comes to assessing cognitive control, the methodologies employed by sleep and hallucinations research fields differ. For example, measurement of ‘attentional cognitive inhibition’ within sleep studies is often done using Stroop tasks (García et al. 2021), whilst ‘intentional inhibition’ within the hallucination field is almost unanimously measured using ‘false alarm’ responses on the Inhibition of Currently Irrelevant Memories (ICIM) task (e.g., Alderson‐Day et al. 2019; Waters et al. 2003). To examine whether cognitive control processes underlie the link between sleep and hallucinations, standardisation of methodology between research fields is required. The current study used a sleep deprivation paradigm in combination with assessments of cognitive control (intentional inhibition, working memory) and an auditory signal detection task (reliance on top‐down processing—often used in hallucination studies due to consistent correlations with measures of hallucinatory experiences) to investigate whether these cognitive processes were impaired in sleep deprived participants (23–25 h) compared to a rested control group across three timepoints (T1/T2/T3). Given the purpose of identifying common mechanisms with hallucinations, and as this study aimed to manipulate sleep, these tasks were selected from those common in the hallucinations literature. The hypotheses presented in Table 1 were pre‐registered (see: doi.org/10.17605/OSF.IO/K7M2Z).

**TABLE 1:** Pre‐registered within‐group and between‐group hypotheses.

H1	Sleep‐deprived participants would show lower performance on cognitive control tasks, compared to controls, at T2 (post‐deprivation)	H1.a	Decreased digit span on the backwards digit span (working memory) task
		H1.b	Increased false alarms on the ICIM (intentional inhibition)
		H1.c	Increased false alarms on the auditory signal detection task
H2	Sleep‐deprived participants would show lower performance on cognitive control tasks at T2 following a period of sleep deprivation, compared to baseline at T1	H2.a	Decreased digit span on the backwards digit span (working memory) task
		H2.b	Increased false alarms on the ICIM (intentional inhibition)
		H2.c	Increased false alarms on the auditory signal detection task

*Note:* Timepoint 1 (T1) = baseline; Timepoint 2 (T2) = post‐sleep deprivation (sleep deprivation group) or habitual sleep (rested control group).

We collected data at a third timepoint (T3: post‐recovery sleep [sleep deprivation group]; post‐habitual activity [rested control group]) and conducted exploratory analysis to examine the extent to which recovery sleep restored cognitive control in the sleep deprivation group compared to the rested control group at T2 and T3.

## 2 Materials and Methods

### 2.1 Design

A 3 × 2 mixed design was used to investigate differences in cognitive control in sleep‐deprived participants compared to rested controls. Independent variables were sleep condition (2 levels between subjects: sleep‐deprived and rested controls) and timepoint (3 levels within subjects: T1, T2, and T3). Dependent variables were performance on cognitive tasks.

### 2.2 Sample

#### 2.2.1 Sample Size Justification

Based on a meta‐analysis on the effects of sleep deprivation on cognition (Lim and Dinges 2010), medium‐to‐large effect sizes were expected. Due to limits on resources, the a priori decision (see pre‐registration: doi.org/10.17605/OSF.IO/K7M2Z) was made to use a more liberal alpha level of *α* = 0.1, which allowed statistical power to remain at 80% and to attempt to balance error rates (Lakens et al. 2018) (exploratory analysis and post hoc tests used a non‐adjusted alpha (*p* < 0.05)). For similar reasons, we opted for an unbalanced design in which participants were recruited into the control group at a 2:1 ratio. Power analysis using G*Power 3.1 (Faul et al. 2007) for the 3 × 2 mixed ANOVA (*f* = 0.35, *α* = 0.1, power = 80%), suggested a target sample size of *N* = 28. For the independent samples *t*‐test (*d* = 0.8, *α* = 0.1, power = 80%, allocation ratio = 2:1) a minimum of *N* = 11 participants in the sleep deprivation group and *N* = 23 participants in the rested control group (*N* = 34) was required. We aimed to recruit a minimum of 15 participants into the sleep deprivation condition, and 30 participants into the control condition, to allow for attrition and data loss.

#### 2.2.2 Participants

Forty‐five participants (female = 15, age *M* = 25.33 years, SD = 3.80) were recruited. Participants were allocated to the experimental sleep deprivation group (*N* = 15; female = 6), or control group (*N* = 30; female = 15) based on their ability to attend the lab for sleep deprivation sessions on dates when testing was possible. Participants from the control group were matched to participants in the sleep deprivation group based on age, gender, income, occupation, education, and substance use (see Supporting Information for demographics breakdown by condition).

Participants were initially randomly allocated to either the sleep deprivation or rested control condition. However, due to external scheduling constraints with the lab, some participants originally assigned to the sleep deprivation condition were unable to attend overnight sessions. These participants were retained on a reserve list and later reassigned as controls if available. Some matching was performed from this reserve list, based on age, gender, income, occupation, education, and substance use to ensure comparability between conditions. Final group composition therefore reflected both random assignment and demographic matching necessary due to scheduling limitations.

The inclusion criteria were to: be between the ages of 18 and 60 years; speak fluent English; and have a stable sleep–wake pattern (i.e., sleep between midnight and 09:00). Participants were excluded if they reported acute or chronic illness, physical, psychological, neurological or sleep disorder; had a diagnosed hearing impairment; drank > 14 units of alcohol per week; or if they were pregnant or seeking to get pregnant.

### 2.3 Materials

#### 2.3.1 Cognitive Tasks

Participants completed the tasks three times (T1/T2/T3). Tasks were programmed using PsychoPy (ICIM, aSDT) and JavaScript (bDST).

*Inhibition of Currently Irrelevant Memories Task (ICIM).* Intentional inhibition was measured using the ICIM task (adapted from Alderson‐Day et al. 2019). The task consisted of three blocks. Black and white images were presented for 2000 ms, with 700 ms between trials. Participants had to judge whether an image was new (key press ‘1’) or a repeat (key press ‘2’) within the current block. In blocks 2 and 3, participants were instructed to ignore (i.e., intentionally inhibit) images from previous blocks. The three blocks (95 trials each) were separated by 30‐s intervals. Stimuli were 60 images: 40 presented only once; 5 presented twice; and 15 presented three times. This allowed for 35 possibilities to correctly identify a repeated image (i.e., ‘hit’) and 60 possibilities to incorrectly classify a first presentation as a repeat (i.e., ‘false alarm’) in each block. As is standard, summed false alarms from blocks two and three were calculated, because these blocks require inhibition of images from block one (Alderson‐Day et al. 2019). New image sets were presented at each timepoint to avoid practise or carryover effects. Spearman‐Brown corrected reliability coefficients revealed high reliability of false alarm responses (Block 1 *r* = 0.97, 95% CIs = [0.93, 1]; summed Block 2 and 3 *r* = 0.95, 95% CIs = [0.93, 0.98]).

*Backwards Digit Span Task (bDST).* The bDST (adapted from Moseley et al. 2021) was used to measure working memory capacity. Participants were presented with 14 trials of a series of digits (ranging from 1 to 9) and were instructed to input the sequence in reverse order, using a mouse‐click on an onscreen keypad. Upon a correct answer, the subsequent trial would be longer by one digit; upon two consecutive incorrect answers, the subsequent trial would be shorter by one digit. Mean digit span was calculated by summing the hit rate for each list length (proportion of correct trials for each list length) with a baseline value of 1.5 (the smallest list length minus 0.5) (Woods et al. 2011).

*Auditory Signal Detection Task (aSDT).* The aSDT assessed the tendency to make false auditory perceptions (Moseley et al. 2021). Participants listened to 72 white noise bursts (3.5 s) through a pair of closed on‐ear headphones (Sennheiser HD 25 Light), with 36 containing a speech clip (1.5 s) at varying signal‐to‐noise ratios (20%, 40%, 60%, 80%), whilst the other 36 trials contained no speech. Speech clips consisted of a monotone male voice reading from an instruction manual. Participants indicated with a button‐press whether they heard a voice in the noise (yes/no) and to state the confidence in their response (not confident at all = ‘1’; very confident = ‘4’). This provided 36 opportunities to correctly identify the presence of a voice (i.e., hit), and 36 opportunities to incorrectly report a voice when none was present (i.e., false alarm). In addition to false alarms and hits, sensitivity ($d^{\prime }=z\left(H\right)-z\left(F\right)$) and response bias ($\beta =e\left\{\frac{Z{\left(\mathrm{FA}\right)}^{2}-Z{\left(H\right)}^{2}}{2}\right\}$) were calculated (Stanislaw and Todorov 1999). This task design followed previous applications (Moseley et al. 2021). The task at baseline showed strong internal consistency (split‐half reliability coefficient *r* = 0.89, 95% CIs = [0.83, 0.94]).

#### 2.3.2 Self‐Report Measures

*Sleep Health Index (SHI).* This was used to measure overall sleep health using 14 items across three subscales: sleep duration, sleep quality, and disordered sleep (Knutson et al. 2017). Raw responses were scored within each subtheme utilising the Sleep Health Index Guidebook and Scoring Manual (The National Sleep Foundation 2017). Each subscale was scored from 0 to 100 with total sleep health as an average of these scores (1 = very poor; 100 = optimum). Previous work has shown good internal consistency and construct validity (Knutson et al. 2017); the current study showed good internal reliability (*α* = 0.71).

*Karolinska Sleepiness Scale (KSS).* The KSS is a single‐item sleepiness Likert measure for self‐perceived alertness or sleepiness (Åkerstedt and Gillberg 1990). The question ‘to what degree do you feel sleepy or alert right now?’ was presented before and after each testing session (extremely alert = ‘1’; very sleepy, a great effort to stay awake, fighting sleep = ‘9’; score range 1–9). This scale is psychometrically valid and consistently correlates with electroencephalographic and behavioural measures of sleepiness (Kaida et al. 2006).

*The Consensus Sleep Diary (CSD).* The CSD is a validated (Dietch and Taylor 2021) participant‐reported measure of subjective sleep (Carney et al. 2012). To attain measurement of overnight sleep, responses allowed for the calculation of their time in bed, total sleep time, sleep latency and onset, as well as overall sleep quality.

*Other measures*. State self‐report measures of negative affect (Positive and Negative Affect Schedule, negative subscale; Watson et al. 1988), intrusive thoughts (Thought Suppression Inventory; Rassin 2003), and emotion regulation (Emotion Regulation Questionnaire; Gross 2002) were also administered at each timepoint. Full descriptions and analyses of these measures can be found in the Supporting Information (doi.org/10.17605/OSF.IO/28SRW).

### 2.4 Procedure

**FIGURE 1:**
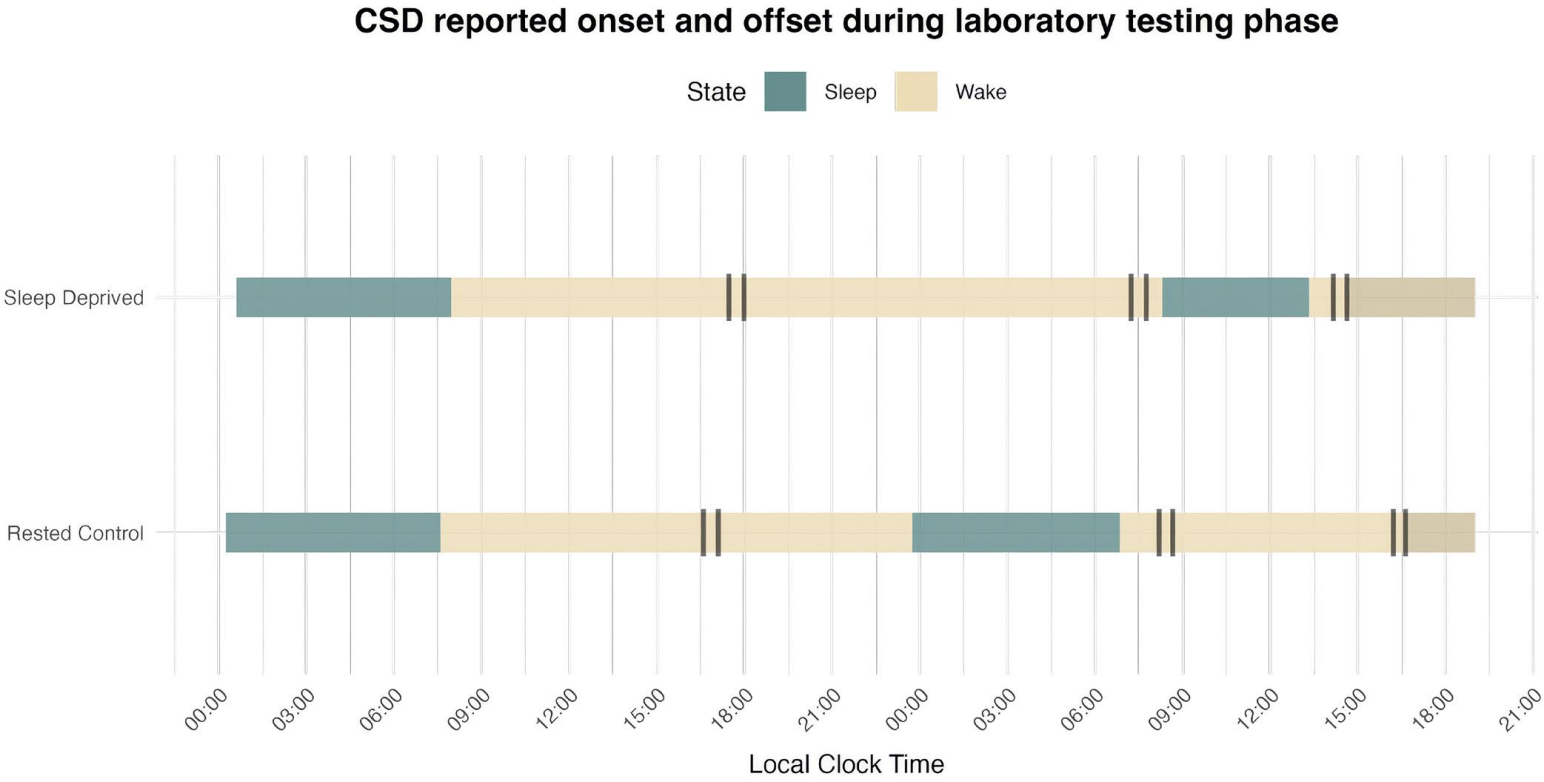
Average sleep onset and offset across groups on lab testing days. *Note:* Vertical black lines indicate the start and end of testing sessions, based on actual mean time tested.

This study received ethical approval from Northumbria University Ethics Committee (ref: 46180). Participants were recruited via campus advertisements and social media. Following informed consent, participants were screened online using an internal medical history questionnaire and the General Health Questionnaire (Goldberg and Blackwell 1970) and for a diagnosis of a sleep disorder using the question, ‘have you ever been diagnosed with, or are you in the process of seeking a diagnosis, for a sleep disorder’ (see Supporting Information). Participants indicated potential sleep disorder symptoms using the Sleep Disorder Symptom Checklist (Klingman et al. 2017), with responses and sleep habits (e.g., average sleep and wake times) discussed in a follow‐up screening to confirm eligibility. They were then allocated to either the sleep deprivation group or the control group depending on both availability and whether they demographically matched a participant from the other group. The protocol timeline is presented in Figure 1. Throughout participation, participants completed the CSD upon wake to assess and compare sleep across groups. Data showed that on average sleep‐deprived participants were awake from 07:58 on the first morning, until 08:18 on the second morning, (duration *M =* 24.3 h), whilst the control group were awake from 07:35 on the first morning to 23:45 that evening (duration *M* = 16.2 h).

On the day of the first testing session, participants were instructed to wake up at their habitual wake time and go about their day as normal. Participants were asked to cease consumption of stimulants (e.g., caffeinated drinks) from the point of baseline testing. Testing periods consisted of completing the KSS and SHI pre‐testing; completing the cognitive tasks (ICIM, aSDT, and bDST); and self‐report measures (TSI, PANAS, and ERQ); and a repeated KSS, post‐testing.

#### 2.4.1 Sleep Deprivation Condition

Baseline testing (T1) was initiated between 16:30 and 17:15. Participants remained awake throughout the night and were allowed to eat or engage in any other activity within the laboratory, except for exercising or sleeping. To remain consistent with the control group in terms of habitual eating, participants brought their own food for dinner and breakfast. Snacks were provided. Post‐sleep deprivation testing (T2) was initiated between 07:30 and 08:00, where participants would have been awake for approximately 23–25 h depending on habitual wake times. After testing, participants began a recovery sleep, with lights‐out no later than 08:30. They were left to sleep for a period of up to 7 h. Upon awakening, participants ate breakfast. Participants then completed post‐recovery sleep testing (T3) approximately 45 min after awakening. Local time for the commencement of T3 was between 13:00 and 15:30, depending on when the individual awoke.

#### 2.4.2 Rested Control Condition

Participants attended the laboratory between 16:30 and 17:15 for baseline testing (T1). They returned home to follow their habitual evening and night routine. Participants returned to the laboratory for testing post‐habitual sleep (T2) between 07:30 and 08:30. Participants then engaged in their habitual daytime routine, before returning for their post‐habitual activity testing session (T3) between 14:30 and 17:00 depending on availability.

Whilst we sought to keep testing times standardised for all participants, there was some variation between the sleep deprivation and control groups in the time between testing sessions, with the control group tending to have slightly longer gaps between sessions, due to variation in arrival times in the lab. Full statistical analysis of time between sessions is presented in the Supporting Information.

### 2.5 Data Analysis

Data was wrangled, cleaned, and analysed in R. Self‐report data was excluded from any single questionnaire measure if the participant completed < 66% of items on that questionnaire; otherwise, missing items were replaced with the mean value of that subscale. ICIM data was excluded if the participant's *d'* (sensitivity) was ≤ 0 (i.e., at or below chance performance); bDST exclusions were made if participants scored < 3 or > 12 (i.e., extremely low or high performance); and aSDT data was removed if the participant scored a *d'* value of ≤ 0, a hit rate of ≤ 10%, or made 100% correct responses (i.e., 100% hit rate, 0% false alarm rate).

ANOVA assumption checks were run for each model. Normality of residuals was checked with Shapiro–Wilk tests and Levene's test for homogeneity of variance. Where normality was violated, square‐root transformations were conducted. This did not improve fit in any circumstances unless otherwise stated. Greenhouse‐Geiser corrections for sphericity were applied to ANOVA models violating the assumption of sphericity (aSDT β). Extreme outliers were identified using boxplot methods, and manual inspection.

The 3 × 2 mixed ANOVAs were conducted using*‘afex’* (Singmann et al. 2023). Condition (sleep deprivation, rested control) was the between‐subjects variable and timepoint (T1, T2, T3) was the within‐subjects variable.

For significant main effects, one‐way repeated‐measures ANOVAs were used to explore within‐group differences, and paired samples *t*‐tests were used for between‐group differences. Bonferroni corrections (*α*_adj_) were applied based on the number of comparisons within each subset of post hoc tests for each variable.

## 3 Results

### 3.1 Self‐Reported Sleepiness

**FIGURE 2:**
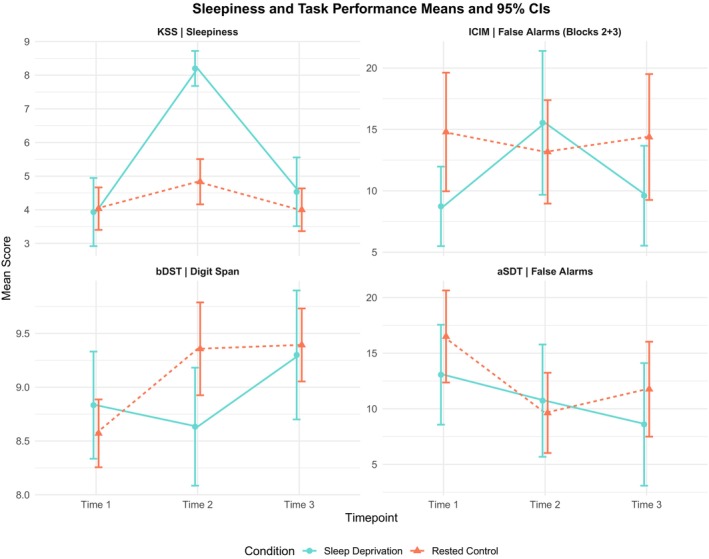
Means and 95% confidence intervals for main outcome measures across conditions and timepoints.

As expected, there was a significant interaction between timepoint and condition on KSS score (see Table 2 and Figure 2). Two one‐way ANOVAs (*α*_adj_, *p* = 0.025) identified an effect of timepoint on KSS score in the SD group, *F*(2, 28) = 43.13, *p* < 0.001, *η*_*p*_^*2*^ = 0.75; however, there was no effect on KSS score over time in the rested control group, *F*(2, 58) = 3.07, *p* = 0.054, *η*_*p*_^*2*^ = 0.10. Independent samples *t*‐tests (*α*_adj_, *p* = 0.017) indicated that the only between‐group difference in sleepiness was at T2 (following sleep deprivation), *t*(42.85) = 8.23, *p* < 0.001, *d* = 2.34, 95% CIs = [1.59, 3.08], with no difference at T3 following the recovery sleep *t*(26.14) = 0.94, *p* = 0.357, *d* = 0.30, 95% CIs = [−0.34, 0.93].

**TABLE 2:** Sleepiness scores and cognitive task performance across timepoints.

	Sleep deprivation group			Rested control group			*F*‐test		
	T1	T2	T3	T1	T2	T3	Condition	Timepoint	Interaction
KSS scores	3.93 (1.83)	8.20^a^ (0.94)	4.53^a^ (1.85)	4.03 (1.69)	4.83^a^ (1.80)	4.00^a^ (1.70)	*F*(1, 43) = 10.88	*F*(2, 86) = 37.25	*F*(2, 86) = 16.47
							*η*_*p*_^*2*^ = 0.20	*η*_*p*_^*2*^ = 0.46	*η*_*p*_^*2*^ = 0.28
							***p* = 0.002**	***p* < 0.001**	***p* < 0.001**
ICIM false alarms (Blocks 2 and 3)	8.73 (5.85)	15.53^a^ (10.57)	9.60^a^ (7.35)	14.79 (12.70)	13.17 (11.08)	14.38 (13.48)	*F*(1, 42) = 0.37	*F*(2, 84) = 1.44	*F*(2, 84) = 7.59
							*η*_*p*_^*2*^ = 0.01	*η*_*p*_^*2*^ = 0.03	*η*_*p*_^*2*^ = 0.15
							*p* = 0.548	*p* = 0.243	***p* < 0.001**
ICIM false alarms (Block 1)	3.53 (4.26)	3.13 (2.67)	2.40 (2.23)	2.21 (2.06)	1.59 (1.50)	3.28^a^ (2.36)	*F*(1, 42) = 1.11	*F*(2, 84) = 1.03	*F*(2, 84) = 5.67
							*η*_*p*_^*2*^ = 0.03	*η*_*p*_^*2*^ = 0.02	*η*_*p*_^*2*^ = 0.12
							*p* = 0.298	*p* = 0.361	***p* = 0.005**
bDST mean span	8.83 (0.90)	8.63 (1.08)	9.30^a^ (1.08)	8.57 (0.81)	9.36 (1.11)	9.39^a^ (0.81)	*F*(1, 41) = 0.52	*F*(2, 82) = 9.68	*F*(2, 82) = 5.80
							*η*_*p*_^*2*^ = 0.01	*η*_*p*_^*2*^ = 0.19	*η*_*p*_^*2*^ = 0.12
							*p* = 0.475	***p* < 0.001**	***p* = 0.004**
aSDT false alarms	13.07 (8.11)	10.73 (9.11)	8.60 (9.95)	16.50 (11.08)	9.63 (9.66)	11.77 (11.42)	*F*(1, 43) = 0.39	*F*(2, 86) = 10.24	*F*(2, 86) = 1.50
							*η*_*p*_^*2*^ = 0.009	*η*_*p*_^*2*^ = 0.19	*η*_*p*_^*2*^ = 0.03
							*p* = 0.536	***p* < 0.001**	*p* = 0.228
aSDT *d'*	1.43 (0.53)	1.35 (0.54)	1.58 (0.65)	1.21 (0.63)	1.55 (0.60)	1.52 (0.77)	*F*(1, 43) = 0.03	*F*(2, 86) = 2.31	*F*(2, 86) = 1.93
							*η*_*p*_^*2*^ < 0.01	*η*_*p*_^*2*^ = 0.05	*η*_*p*_^*2*^ = 0.04
							*p* = 0.875	*p* = 0.105	*p* = 0.151
aSDT (β)	1.40 (1.71)	2.20 (2.21)	3.49 (4.02)	1.10 (1.56)	1.80 (1.52)	2.53 (3.04)	*F*(1, 43) = 0.79	*F*(1.44, 61.97) = 10.47	*F*(1.44, 61.97) = 0.42
							*η*_*p*_^*2*^ = 0.02	*η*_*p*_^*2*^ = 0.20	*η*_*p*_^*2*^ < 0.01
							*p* = 0.378	***p* < 0.001**	*p* = 0.590

Abbreviations: aSDT, auditory signal detection task; bDST, backwards digit span task; ICIM, intentional inhibition of currently irrelevant memories; KSS score, Karolinska sleepiness scale.

^a^ Scores significantly differ from previous within‐group timepoint.

### 3.2 The Effect of Sleep Deprivation on Intentional Inhibition (ICIM Task)

One control participant was removed from the analysis due to missing data from one timepoint.

There was a significant interaction between condition and timepoint for false alarms in Blocks 2 and 3 of the ICIM (see Table 2 and Figure 2). There was no effect of timepoint (*α*_adj_, *p* = 0.050) within the rested control group, *F*(2, 56) = 1.97, *p* = 0.149, *η*_*p*_^*2*^ = 0.07. Within the sleep deprivation group, repeated‐measures ANOVAs found that false alarms significantly differed over time, *F*(2, 28) = 5.22, *p* = 0.012, *η*_*p*_^*2*^ = 0.27. Supportive of hypothesis H2.b, paired samples *t*‐tests (*α*_adj_, *p* = 0.033) found significantly more false alarms on the ICIM at T2 than T1, *t*(14) = 2.85, *p* = 0.013, *d* = 0.74, 95% CIs = [0.15, 1.30], with significantly fewer false alarms at T3 compared to T2, *t*(14) = −2.59, *p* = 0.021, *d* = −0.67, 95% CIs = [−1.22, −0.10]. There were no differences in the sleep deprivation group at T1 compared to T3, *t*(14) = −0.12, *p* = 0.904, *d* = −0.03, 95% CIs = [−0.54, 0.48]. This suggests an increase in false alarms following sleep deprivation, which decreased to baseline following a recovery sleep.

Independent samples *t‐*tests (*α*_adj_, *p* = 0.025) indicated no significant difference in the number of false alarms made by the rested control versus the sleep deprivation group at T1, *t*(41.77) = −1.50, *p* = 0.141, *d* = −0.43, 95% CIs = [−1.01, 0.14], or T3, *t*(40.89) = −1.63, *p* = 0.111, *d* = −0.48, 95% CIs = [−1.06, 0.11]. In contrast to hypothesis H1.b, there was no significant group difference in false alarms at T2, *t*(27.21) = 1.00, *p* = 0.328, *d* = 0.32, 95% CIs = [−0.32, 0.95]. Therefore, whilst differing within‐group patterns across time were observed, between‐group differences were not observed at individual time points.

In contrast to Blocks 2 and 3, repeated measures ANOVAs (*α*_adj_, *p* = 0.025) on Block 1 false alarms found no effect of timepoint in the sleep deprivation group, *F*(2, 28) = 1.02, *p* = 0.375, *η*_*p*_^*2*^ = 0.07, but a significant effect of timepoint on scores in the rested control group, *F*(2, 56) = 9.39, *p* < 0.001, *η*_*p*_^*2*^ = 0.25. Within the rested control group, paired samples *t‐*tests (*α*_adj_, *p* = 0.017) found no difference in Block 1 false alarms between T1 and T2, *t*(28) = −1.61, *p* = 0.119, *d* = −0.30, 95% CIs = [−0.67, 0.08], but there was a difference at T2 compared to T3, *t*(28) = 5.31, *p* < 0.001, *d* = 0.99, 95% CIs = [0.53, 1.43]. There was no difference in Block 1 false alarms between T1 and T3, *t*(28) = 2.30, *p* = 0.029, *d* = 0.43, 95% CIs = [0.04, 0.80].

### 3.3 The Effect of Sleep Deprivation on Working Memory (bDST)

Two control participants were removed from the analysis due to missing data files.

There was a significant interaction between condition and timepoint for mean span (see Table 2 and Figure 2). Further analysis using repeated measures ANOVAs (*α*_adj_, *p* = 0.050) found an effect of timepoint in the sleep deprivation group, *F*(2, 28) = 4.41, *p* = 0.022, *η*_*p*_^*2*^ = 0.24, and the rested control group, *F*(2, 54) = 14.01, *p* < 0.001, *η*_*p*_^*2*^ = 0.34. In the rested control group (*α*_adj_, *p* = 0.033), mean span was significantly greater at T2 than T1, *t*(27) = 4.53, *p* < 0.001, *d* = 0.86, 95% CIs = [0.42, 1.29], but there was no difference in mean span at T2 compared to T3, *t*(27) = −0.19, *p* = 0.851, *d* = −0.04, 95% CIs = [−0.41, 0.34]. There was a significant difference between T1 and T3, *t*(27) = 5.04, *p* < 0.001, *d* = 0.95, 95% CIs = [0.50, 1.39]. This is suggestive of a general increase in mean span with repeated task completion in the rested control group. Pairwise comparisons (*α*_adj_, *p* = 0.033) found, in contrast to hypothesis H2.a, there was no difference between mean span in the sleep deprivation group at T1 compared to T2, *t*(14) = 0.90, *p* = 0.384, *d* = 0.23, 95% CIs = [−0.29, 0.74]. However, within the sleep deprivation group at T3 (following recovery sleep), mean span significantly increased from T2, *t*(14) = −2.65, *p* = 0.019, *d* = −0.68, 95% CIs = [−1.24, −0.11]. There was no difference between T1 and T3, *t*(14) = −2.17, *p* = 0.048, *d* = −0.56, 95% CIs = [−1.10, −0.01]. In contrast to the rested control group, there was therefore no increase in mean span with practise following sleep deprivation; however, following recovery sleep, there was an improvement with further task completion.

In contrast to H1.a, independent samples *t‐*tests (*α*_adj_, *p* = 0.033) comparing rested control and sleep deprivation mean span across timepoint showed no significant difference (at the corrected alpha level) at T2, *t*(31.80) = −2.19, *p* = 0.036, *d* = −0.69, 95% CIs = [−1.32, −0.04]. There was also no difference at the other timepoints: T1, *t*(26.32) = 0.94, *p* = 0.356, *d* = 0.31, 95% CIs = [−0.34, 0.94]; T3, *t*(24) = −0.29, *p* = 0.777, *d* = −0.09, 95% CIs = [−0.74, 0.55].

### 3.4 The Effect of Sleep Deprivation on Top‐Down Processing (aSDT)

The data was not normally distributed for false alarms or response bias (β) scores on the aSDT. Square‐root transformations improved normality for false alarms only; therefore, transformed variables were used for this variable, whilst non‐transformed variables were used for all others.

There was no interaction between condition and group for any of the aSDT variables (false alarms, $d^{\prime }$, β). Whole sample comparisons indicated a decrease in false alarms on repeated task completion, linked to an increase in β across timepoints—but this did not differ between sleep‐deprived and control participants (see Table 2, Figure 2, and Supporting Information, for inferential statistics).

### 3.5 Self‐Reported Affect, Thought Suppression, and Emotion Regulation

Participants completed self‐report measures aimed at monitoring negative affect, intrusive thoughts, and emotion regulation at the three timepoints. There were no differences between conditions or timepoints in TSI, PANAS‐NA, or ERQ scores (see Supporting Information).

## 4 Discussion

The current study aimed to investigate the effects of sleep deprivation on cognitive control mechanisms in a general population sample using a 23–25 h total sleep deprivation paradigm. To summarise, sleep deprivation impacted intentional inhibition and working memory capacity. In line with the second hypothesis, lapses in intentional inhibition were more frequent post‐sleep deprivation compared to baseline and returned to levels similar to baseline following recovery sleep. In contrast, there was no change in the control group's intentional inhibition ability across timepoints. Contrary to the first hypothesis, no significant difference was found between groups in intentional inhibition ability at T2.

These findings suggest sleep deprivation impairs the ability to inhibit irrelevant information from memory, which aligns with research linking sleep loss to impaired inhibitory control contributing to reduced suppression of irrelevant or inappropriate responses, and non‐goal relevant stimuli in working memory (Anderson and Platten 2011; Aron 2007; Zhao et al. 2019). Recovery sleep appears to reverse this impairment, supporting the idea that increased biological sleep pressure may affect inhibitory control. Previous work has indicated higher sleep pressure, elicited by partial sleep restriction or total sleep deprivation, has been seen to impair inhibition control on response inhibition tasks and is associated with a decline in vigilant attention (Mao et al. 2021). To our knowledge, this is the first demonstration of impaired inhibition following sleep deprivation using the ICIM task, performance on which has been associated with hallucinations in psychosis (Waters et al. 2003).

It could be argued that these results can be explained by the effect of sleep deprivation on mnemonic abilities more broadly. However, there was no change in performance due to sleep deprivation on the first block of the ICIM task (a standard continuous recognition paradigm, with no instructions regarding inhibition), indicating that specific memory processes, such as controlled memory inhibition rather than more general memory are particularly affected by sleep deprivation.

Sleep deprivation had mixed effects on working memory task performance. There was no difference between working memory scores at T2 in the sleep deprivation group compared to the rested control group. However, whilst the rested control group showed a general increase in working memory performance with repeated task completion, the sleep deprivation group did not show this pattern until after a recovery sleep (T3) suggesting that, whilst performance was not reduced by sleep deprivation, improvement in task performance was inhibited. These findings indicate sleep works to maintain working memory capacity, supporting prior research reporting that sleep, napping, and sleep characteristics such as slow‐oscillatory brain activity enhance working memory performance (Könen et al. 2015; Sattari et al. 2019).

Contrary to our hypotheses, there was no observable effect of sleep deprivation on auditory signal detection task performance, on any of the signal detection parameters, suggesting that signal detection processes remain robust against the perturbations induced by sleep deprivation. It has been previously argued that this task assesses reliance on top‐down processes—our results suggest that sleep deprivation does not affect top‐down processing on this task, and hence may not underlie atypical top‐down processing in psychosis.

Taken together, these results may suggest that the association between sleep dysfunction and hallucinations may be related to impaired intentional inhibition, but not auditory top‐down processing. These findings provide tentative support for Harrington and Cairney's (2021) theoretical proposition that sleep loss impairs the ability to control unwanted thoughts, for example due to weakened inhibitory projections from the right dorsolateral prefrontal cortex (rDLPFC), which orchestrates memory suppression within regions like the hippocampus and amygdala (Harrington et al. 2021). Diminished activity in the rDLPFC is associated with impaired inhibitory control, which attenuates the suppression of unwanted thoughts, and is exacerbated by cognitive or mental fatigue (Kayser et al. 2022). Furthermore, sleep deprivation leads to failures in top‐down memory control (Zhao et al. 2019), which contributes to difficulty suppressing intrusive thoughts or memories (Gagnepain et al. 2017). When sleep‐deprived, both the content and affective tone of the memory intrude into consciousness, causing emotion dysregulation (Harrington and Cairney 2021). Whilst the current study found that working memory and intentional inhibition were impacted by sleep loss, it should be noted that there was no observable effect on self‐reported thought control or emotion regulation (see Supporting Information).

Regardless, the current study is evidence that sleep deprivation impairs some cognitive control mechanisms associated with hallucinations, although it appears not to affect performance on tasks associated with auditory top‐down processing.

### 4.1 Limitations and Future Research

There were several limitations to consider. Firstly, availability constraints meant rested control participants could not remain in the laboratory overnight, which likely influenced variation in duration between testing timepoints across groups. It is unlikely that this difference caused the effects observed, firstly because the sleep deprivation manipulation was effective (supported by KSS results), and secondly because, in general, rested control task performance improved at T2 despite expected attenuation of practise effects. Participant group allocation was initially random, yet practical constraints related to lab scheduling meant this could not be maintained. Some participants from the reserve list were recruited as controls to match available sleep deprivation participants on key demographics (age, gender, income, occupation, education, and substance use). Whilst matching helped preserve group comparability, the final allocation process may have introduced selection bias between groups.

The current sample was predominantly students, White, and with income needs met. Psychotic disorders and positive symptoms, such as hallucinations, are more common in ethnic minority groups (Jongsma et al. 2021) and in populations with lower socioeconomic class (Murali and Oyebode 2004). Thus, it is possible these findings may not be generalisable to populations with psychosis, warranting further research to investigate individual differences in sleep deprivation.

Finally, whilst our sample is typical for sleep deprivation research (Lim and Dinges 2010; Waters et al. 2018) it does mean that the frequency of Type II errors (i.e., false negatives) may be high for small or medium effect sizes. To mitigate this, we relaxed our alpha level (alpha = 0.10), at the expense of an increased Type I error rate (i.e., more false positives). However, none of the conclusions were affected by this decision, with all pre‐registered tests falling below the traditional alpha level of 0.05. Future research should aim to recruit larger sample sizes, potentially using multi‐site approaches.

Building on findings that support neurocognitive models of intrusive thoughts and memory control (Harrington and Cairney 2021), future research should explore the role of the rDLPFC in inhibiting unwanted thoughts and emotions. Understanding this relationship may provide insight into related psychotic symptoms, such as hallucinations, and help develop treatments that target cognitive control. Additionally, identifying which cognitive control mechanisms are most vulnerable to sleep deprivation can inform hypotheses about the role of sleep in psychopathological features like hallucinations.

### 4.2 Conclusions

This study aimed to examine the effects of sleep deprivation on cognitive control mechanisms associated with hallucinations. The findings indicate that sleep deprivation impairs intentional inhibition (but not continuous recognition), and therefore suggest an impact of sleep deprivation on conscious inhibitory cognitive control processes. Given the known link between sleep dysfunction and psychopathology, understanding the role of cognitive control is paramount for the development of interventions to mitigate the effects of sleep loss and symptoms of mental health disorders, such as hallucinations.

## Author Contributions

G.P. designed the study methodology, led ethics and data collection, conducted all statistical analyses, and managed manuscript preparation, editing, and submission. J.G.E. supervised ethics submission and manuscript editing, and provided guidance on study design, methodology, and theory. C.M., E.J., M.T., and F.S., contributed to methodological guidance, data collection and manuscript editing. D.S. supervised data interpretation and manuscript editing. P.M. supervised study design, ethics, verified statistical analysis, contributed to data interpretation, and manuscript editing.

## Funding

The authors have nothing to report.

## Disclosure

The authors have nothing to report.

## Ethics Statement

This study received ethical approval from Northumbria University Ethics Committee (ref: 46180).

## Conflicts of Interest

The authors declare no conflicts of interest.

## Supporting information


**Data S1:** Supporting Information.

## Data Availability

The data that support the findings of this study are openly available on the Open Science Framework at https://osf.io/28srw/, reference number DOI 10.17605/OSF.IO/28SRW.
