# Supplementary material for: The Effects of Acute Sleep Deprivation on Cognitive Control Mechanisms Associated With Hallucinatory Experiences
Source: J Sleep Res. 2025 Dec 9;35(3):e70259. doi: 10.1111/jsr.70259 (PMC13193439; doi:10.1111/jsr.70259)
Supplement: Supplementary file 1 — Data S1: Supporting Information. [file JSR-35-e70259-s001.docx]

The effects of acute sleep deprivation on cognitive control mechanisms associated with hallucinatory experiences

Supplementary Materials

Methods

Supplementary Table 1 contains additional information on participant demographics.

Supplementary Table 1

*Demographic information for sleep deprivation and rested control groups*

| Variable | Total  *N* = 45 | Sleep Deprivation  *N* = 15 | | | Rested Control  *N* = 30 | |
| --- | --- | --- | --- | --- | --- | --- |
|  |  | N | % | | N | % |
| Age (mean, SD) | 25.33 (3.80) | 24.33 (1.68) | | | 25.83 (4.45) | |
| Sleep Health (mean, SD)* | 74.04 (12.06) | 76.28 (12.96) | | | 72.92 (11.65) | |
| Sex  Male  Female | 24  21 | 9  6 | | 60  40 | 15  15 | 50  50 |
| Ethnic Group  White  Asian or Asian British | 44  1 | 15  0 | | 100  0 | 29  1 | 96.67  3.33 |
| Completed Education  A-Level  Foundation  Undergraduate  Postgraduate  Doctorate | 8  4  10  20  3 | 3  0  0  11  1 | | 20  0  0  73.33  6.67 | 5  4  10  9  2 | 16.67  13.33  33.33  30  6.67 |
| Income  Less than enough  Enough  More than enough | 1  36  8 | 0  13  2 | | 0  86.67  13.33 | 1  23  6 | 3.33  76.67  20 |
| Alcohol  Never  Monthly or less  2-4 times per month  2-3 times per week  4+ times per week | 4  15  21  3  2 | 0  5  8  1  1 | | 0  33.33  53.33  6.67  6.67 | 4  10  13  2  1 | 3.33  33.33  43.33  6.66  3.33 |
| Nicotine  Never  Monthly or less  2-4 times per month  2-3 times per week  4+ times per week | 30  3  3  3  6 | 7  3  1  1  3 | | 46.67  20  6.67  6.67  20 | 23  0  0  2  3 | 76.67  0  0  6.66  10 |

*Note.* All participants identified as cisgender, with their gender aligning with assigned sex-at-birth. *No significant difference in SHI score between groups, *t*(26.80) = .88, *p* = .384.

The average time between testing session was longer in the rested control group between baseline testing and post-habitual sleep (minutes; *M* = 930.80, *SD* = 46.95) compared to participants in the sleep deprivation group between baseline and post-sleep deprivation (minutes; *M* = 836.00, *SD* = 30.71), *t*(39.68) = -8.12, *p* < .001, *d* = -2.24, 95% CIs = [-3.01, -1.45]; and the duration between testing for the rested control group between the post-habitual sleep testing session and the post-habitual activity testing session (minutes; mean = 483.80, SD = 56.91) was also longer than the duration between the sleep deprivation group post-sleep deprivation testing session (minutes; mean = 404.87, SD = 47.28) and post-recovery sleep testing session, *t*(33.21) = -4.92, *p* < .001, *d* = -1.46, 95% CIs = [-2.15, -0.76]. This was mainly due to variation on arrival times to the testing session T2 and T3.

**Measures**

**Trait measure of baseline hallucinatory experiences**

*Multi-Modality Unusual Sensory Experiences Questionnaire (MUSEQ).* To measure hallucinatory experiences of all sensory modalities, the 43-item trait assessment, the MUSEQ (Mitchell et al., 2017), was used. Six subscales assess the frequency of unusual sensory experiences of each sensory modality: auditory, visual, olfactory, gustatory, bodily sensations, and sensed presence (e.g., ‘my ears have played tricks on me’; ‘I have seen lights, flashes, or other shapes that other people could not see’). Responses were indicated using Likert-style responses (0 = ‘never: never happened’, 4 = ‘frequently: at least monthly’). Overall scores were the sum of all items in all subscales, with scores ranging from 0 to 172 with higher scores indicating a greater frequency of hallucinatory experiences. Each subscale presented good internal consistency (*α* = .77 - .88), acceptable test-retest reliability (*r* = .56 - .72) and good convergent and divergent validity (Mitchell et al., 2017). Internal consistency was considered excellent (*α* = .97).

Mean MUSEQ total scores were slightly higher in the sleep deprivation group (M = 49.27, SD = 30.10, n = 15) compared with the rested control group (M = 39.53, SD = 24.86, n = 30), and both samples had scores lower than previous studies testing in non-clinical groups (Mitchell et al., 2017). An independent-samples t-test indicated that this difference was not statistically significant, t(43) = 1.15, p = .26, 95% CI [–7.28, 26.75]. These results suggest that the occurrence of hallucinatory experiences did not differ across groups.

**Self-report measures of thought suppression and emotion regulation**

State self-report measures were given at each timepoint, with participants instructed to report based on their experience over the last few hours, or since their last testing session. The Thought Suppression Inventory and Emotion Regulation Questionnaire were both adapted to obtain responses relevant to participants states ‘over the last few hours’ (see supplementary materials; ).

*Thought Suppression Inventory (TSI).* The 15-item TSI measured general control over intrusive thoughts[44] using summed scores of Likert responses (i.e., 1 = strongly disagree, 5 = strongly agree; subscale range 5 – 25) on three separate subscales: ‘intrusions’ (e.g., ‘I have many unpleasant thoughts'), ‘suppression attempts’ (e.g., ‘I try to avoid certain thoughts’), and ‘successful suppression’ (e.g., ‘I am able to put aside problems and worries’). Higher scores indicated a greater frequency of effort to control and/or successfully suppress intrusive thoughts. The TSI had been found to be psychometrically sound for use to assess these three dimensions[45]. At baseline within the current study two subscales were acceptable (‘suppression attempts’, *α* = .67; ‘successful suppressions’, *α* = .79), however, the ‘intrusions’ subscale had low reliability (*α* = .47) due to one item. However, once removed the pattern of results did not change, therefore the original ‘intrusions’ subscale was reported.

*Emotion Regulation Questionnaire (ERQ).* To assess emotion regulation strategies, the 10-item ERQ was employed [46]. The two subscale outcome measures represent emotion regulation strategies: ‘expressive suppression’ (e.g., ‘I keep my emotions to myself’) and ‘cognitive reappraisal’ (e.g., ‘I control my emotions by changing the way I think about the situation I’m in’), as calculated by summing Likert responses on a 7-point scale (i.e., 1 = strongly disagree, 7 = strongly agree; cognitive reappraisal range 6 – 42, expressive suppression range 4 – 24). Higher scores indicated greater use of the respective strategy. This questionnaire has strong psychometric properties when used in general population samples [47], and internal consistency within the current study was high at baseline testing. (*α* = .84, ‘expressive suppression’; *α* = .83 ‘cognitive reappraisal’).

*Positive and Negative Affect Scales – Negative Affect (PANAS-NA).* Negative affect was measured using the 10-item negative affect subscale of the PANAS [48]. The outcome variable was total summed scores of the 5-point Likert responses (i.e., 1 = very slightly or not at all, 5 = extremely; scale range 10 – 50). Higher scores indicated greater negative affect. This measure is valid and reliable for use in the general population [48,49], and in the current study had high internal consistency at baseline (*α* = .88).

**Expanded Results**

***Further analysis of task performance between groups***

***ICIM: Hits.*** A 2 x 3 mixed ANOVA of the ICIM-Hits variable (i.e., correctly identified repeats) found a significant effect of timepoint, *F*(2, 84) = 12.40, *p* < .001, *η_p_^2^* < .23, but no main effect of condition, *F*(1, 42) = .15, *p* = .705, *η_p_^2^* = .003, or interaction between condition and timepoint on ICIM-Hits, *F*(2, 84) = .82, *p* = .446, *η_p_^2^* = .02, indicating that ICIM-Hits do not significantly differ between the SD and RC conditions across timepoints.

Full sample pairwise comparisons (*α*_adj_, *p* = .017) determined ICIM-Hits decreased from T1 to T2, *t*(43) = -4.07, *p* = < .001, *d* = -.61, 95% CIs = [-.93, -.29], and remained reduced in T3 compared to T1, *t*(43) = -4.49, *p* = < .001, *d* = -.68, 95% CIs = [-1.00, -.35]. There was no difference between ICIM-Hits at T2 compared to T3, *t*(43) = -.90, *p* = .372, *d* = -.14, 95% CIs = [-.43, .16].

***aSDT: Hits.*** A 2 x 3 ANOVA investigating aSDT-Hits (i.e., correctly identified voices) as the dependent variable found no main effect of condition, *F*(1, 43) = .2.36, *p* = .131, *η_p_^2^* = .05. There was a main effect of timepoint, *F*(2, 86) = 17.13, *p* = < .001, *η_p_^2^* = .28, but no condition x timepoint interaction, *F*(2, 86) = .81, *p* = .447, *η_p_^2^* = .02.

Whole sample comparisons (*α*_adj_, *p* = .017) found a reduction in aSDT-Hits between T1 and T2, *t*(44) = -5.16, *p* < .001, *d* = -.77, 95% CIs = [-1.10, -.43], and T1 and T3, *t*(44) = -4.37, *p* < .001, *d* = -.65, 95% CIs = [-.97, -.33]. No difference was observed between T2 and T3, *t*(44) = 1.06, *p* = .296, *d* = .16, 95% CIs = [-.14, .45].

Supplementary Table 2

*Descriptive statistics of all task variables entered into 2 x 3 mixed ANOVAs*

| Condition | Timepoint |  | | | | | | | |
| --- | --- | --- | --- | --- | --- | --- | --- | --- | --- |
|  |  | **ICIM-FA*** | ICIM-Hits | **ICIM-B1** | **bDST-MS*** | aSDT-FA* | aSDT-Hits | aSDT-d′ | aSDT-beta |
| Sleep Deprivation | 1  2  3 | **12.27**  **(7.41)**  **18.67**  **(12.53)**  **12.00**  **(8.63)** | 55.20  (7.08)  48.87  (12.86)  49.53  (16.53) | **3.53**  **(4.26)**  **3.13**  **(2.67)**  **2.40**  **(2.23)** | **8.83**  **(.90)**  **8.63**  **(1.08)**  **9.30**  **(1.08)** | 13.07  (8.11)  10.73  (9.11)  8.60  (9.95) | 29.20  (3.43)  25.40  (5.29)  25.53  (5.79) | 1.43  (.53)  1.35  (.54)  1.58  (.65) | 1.40  (1.71)  2.20  (2.21)  3.49  (4.02) |
| Rested Control | 1  2  3 | **17.00**  **(13.5)**  **14.76**  **(11.93)**  **17.66**  **(14.36)** | 55.37  (9.81)  51.86  (9.22)  49.96  (10.30) | **2.21**  **(2.06)**  **1.59**  **(1.50)**  **3.28**  **(2.36)** | **8.57**  **(.81)**  **9.36**  **(1.11)**  **9.39**  **(.88)** | 16.50  (11.08)  9.63  (9.66)  11.77  (11.42) | 30.30  (4.17)  27.43  (4.21)  28.20  (4.74) | 1.21  (.63)  1.55  (.60)  1.52  (.77) | 1.10  (1.56)  1.80  (1.52)  2.53  (3.04) |

*Note.* * Pre-registered hypotheses. Bold columns = 2 x 3 interaction was significant. Contains summed ICIM false alarms and hits (summed Block 2 and Block 3); ICIM false alarms from Block 1 only; bDST-MS; raw aSDT false alarms, hits and signal detection parameters (sensitivity [d-prime], and response bias [beta]).

Supplementary Table 3

*Descriptive statistics of all exploratory self-report variables entered into 2 x 3 mixed ANOVAs*

| Condition | Timepoint | Mean (SD) | | | | | | |
| --- | --- | --- | --- | --- | --- | --- | --- | --- |
|  |  | TSI-Int | TSI-SA | TSI-SS | PANAS | ERQ-R | **ERQ-S** | KSS |
| Sleep Deprivation | 1  2  3 | 10.40  (3.56)  12.13  (4.56)  10.53  (3.98) | 16.27  (3.71)  16.20  (4.21)  15.80  (3.45) | 17.47  (4.00)  17.80  (4.55)  18.20  (2.54) | 14.27  (5.20)  14.47  (5.50)  13.67  (4.53) | 31.73  (5.56)  31.07  (5.22)  33.33  (4.29) | **23.67**  **(6.21)**  **20.67**  **(5.90)**  **22.40**  **(4.93)** | 3.93  (1.83)  8.20  (.94)  4.53  (1.85) |
| Rested Control | 1  2  3 | 10.53  (2.80)  9.77  (3.18)  9.70  (2.94) | 17.40  (3.56)  17.27  (3.88)  18.12  (3.12) | 16.83  (4.07)  16.63  (4.12)  17.36  (3.64) | 15.40  (6.04)  13.03  (3.29)  13.43  (3.95) | 33.63  (5.60)  34.57  (3.89)  34.30  (3.91) | **22.70**  **(5.52)**  **23.50**  **(4.69)**  **23.10**  **(6.31)** | 4.03  (1.69)  4.83  (1.80)  4.00  (1.70) |

*Note.* All variables presented here were exploratory. Bold columns = 2 x 3 interaction was significant. TSI intrusions, suppression attempts, and successful suppression; PANAS negative affect; ERQ reappraisal and suppression; KSS scores pre-testing period.

### The effect of sleep deprivation on top-down processing (aSDT)

Assumptions checks showed that the data was not normally distributed for false alarms or response bias ($\beta$) scores on the aSDT. Square root log transformations improved model fit for false alarms only; therefore, transformed variables were used for this variable, while non-transformed variables were used for all others.

There was no interaction between condition and group for aSDT false alarms, though there was an effect of timepoint (see Table 2). Whole sample comparisons (*α*_adj_, *p* = .033) found a significant decrease in false alarms between T1 and T2, *t*(44) = -3.90, *p* < .001, *d* = -.58, 95% CIs = [-.89, -.26], and T1 and T3, *t*(44) = -4.40, *p* < .001, *d* = -.66, 95% CIs = [-.98, -.33]. There was no difference between T2 and T3, *t*(44) = -.44, *p* = .661, *d* = -.07, 95% CIs = [-.36, .23].

We also explored the effects of sleep deprivation on signal detection parameters for sensitivity ($d^{'})$and response bias ($\beta)$ using 3 x 2 ANOVAs. There were no significant effects of condition or timepoint, nor interactions between the two, for these parameters, with the exception of an increase in response bias across both conditions with repeated task completion (see Table 2). Whole sample comparisons (*α*_adj_, *p* = .017) found that response bias was observed to be greater at T2 compared to T1, *t*(44) = 3.18, *p* = .003, *d* = .47, 95% CIs = [.16, .78], and a greater difference was observed at T3 compared to T1, *t*(44) = 3.76, *p* < .001, *d* = .56, 95% CIs = [.24, .87]. However, the difference between T2 and T3 was not significant (at the corrected alpha level), *t*(44) = 2.39, *p* = .021, *d* =.36, 95% CIs = [.05, .66].

### The effect of sleep deprivation on intrusive thoughts and suppression

***Intrusions.*** For the intrusions subscale of the TSI, the 2 x 3 mixed ANOVA revealed no significant main effect of condition, *F*(1, 43) = 1.32, *p* = .257, *η_p_^2^* = .03, nor was there a main effect of timepoint on intrusions, *F*(2, 43) = 1.37, *p* = .260, *η_p_^2^* = .03. There was a significant interaction between condition and timepoint, *F*(2, 86) = 3.11, *p* = .0497, *η_p_^2^* = .07, signifying a difference in intrusions between SD and RC groups across the three timepoints, however when Bonferroni corrections were applied (*α*_adj_, *p* = .017), this was no longer deemed significant.

Further repeated measures one-way ANOVAs for each group intrusion scores across timepoints were conducted (*α*_adj_, *p* = .025). There was no effect of timepoint in the SD group, *F*(2, 28) = 1.84, *p* = .178, *η_p_^2^* = .12, or in the RC group, *F*(2, 58) = 1.64, *p* = .202, *η_p_^2^* = .05.

***Suppression attempts.*** The 2 x 3 mixed ANOVA for the suppression attempts subscale of the TSI found no significant main effect of condition, *F*(1, 43) = 2.74, *p* = .105, *η_p_^2^* = .06, or timepoint *F*(2, 86) = .08, *p* = .927, *η_p_^2^* = .002. There was also no interaction effect found for this variable between condition and timepoint, *F*(2, 86) = .71, *p* = .476, *η_p_^2^* = .02.

***Successful suppression.*** The 2 x 3 mixed ANOVA for the successful suppression subscale of the TSI found no significant main effect of condition, *F*(1, 43) = .64, *p* = .429, *η_p_^2^* = .01, or timepoint*, F*(2, 86) = 1.03, *p* = .361, *η_p_^2^* = .02, and there was also no interaction effect found between condition and timepoint, *F*(2, 86) = .15, *p* = 857, *η_p_^2^* = .004

### The effect of sleep deprivation on negative affect

There were two extreme outliers, but after reviewing the residuals, these were not deemed necessary for removal.

The 2 x 3 mixed ANOVA found there was no significant main effect of condition on PANAS-NA scores, *F*(1, 43) = .02, *p* = .894, *η_p_^2^* < .001. There was no effect of timepoint, *F*(2, 86) = 2.60, *p* = .080, *η_p_^2^* = .06. Additionally, there was no interaction effect between condition and timepoint on PANAS-NA scores, *F*(2, 86) = 2.25, *p* = .111, *η_p_^2^* = .05.

### The effect of sleep deprivation on emotion regulation strategies

***Cognitive reappraisal.*** The 2 x 3 mixed ANOVA for ERQ reappraisal found there was no significant main effect of condition, *F*(1, 43) = 2.77, *p* = .103, *η_p_^2^* = .06, and no main effect of timepoint, *F*(2, 86) = 1.73, *p* = .184, *η_p_^2^* = .04. Additionally, there was no interaction effect between condition and timepoint on reappraisal scores, *F*(2, 86) = 1.85, *p* = .164, *η_p_^2^* = .04.

***Expressive suppression.*** The 2 x 3 mixed ANOVA found there was no significant main effect of condition on ERQ expressive suppression scores, *F*(1, 43) = .27, *p* = .605, *η_p_^2^* = .006, and no main effect of timepoint, *F*(2, 86) = 1.86, *p* = .162, *η_p_^2^* = .04. There was an interaction effect between condition and timepoint on ERQ expressive suppression scores, *F*(2, 86) = 5.51, *p* = .006, *η_p_^2^* = .11, indicating a significant difference in SD and RC expressive suppression scores across timepoints.

Repeated measures one-way ANOVAs (*α*_adj_, *p* = .025) found an effect of timepoint in the SD group, *F*(2, 28) = 4.45, *p* = .021, *η_p_^2^* = .24, but not in the RC group, *F*(2, 58) = .79, *p* = .459, *η_p_^2^* = .03.

Paired samples *t-*tests (*α*_adj_, *p* = .017) were conducted for the SD condition to further compare scores between timepoints emotion suppression did not significantly differ at T2 compared to T1, *t*(14) = -2.36, *p* = .033, *d* = -.61, 95% CIs = [-1.15, -.05], no difference was observed between T2 and T3 scores, *t*(14) = 1.89, *p* = .080, *d* = .49, 95% CIs = [-.06, 1.02], or between scores at T1 and post-recovery T3, *t*(14) = -1.64, *p* = .123, *d* = -.42, 95% CIs = [-.95, .11].
